# The Genetic Polymorphisms and Colonization Process of Olive Fly Populations in Turkey

**DOI:** 10.1371/journal.pone.0056067

**Published:** 2013-02-14

**Authors:** Ersin Dogaç, İrfan Kandemir, Vatan Taskin

**Affiliations:** 1 Department of Biology, Faculty of Science, Muğla Sitki Kocman University,Muğla, Turkey; 2 Department of Biology, Faculty of Science, Ankara University, Ankara, Turkey; University of Sydney, United States of America

## Abstract

The olive fruit fly, *Bactrocera oleae*, is the most important pest of olives in olive growing regions worldwide, especially in the Mediterranean basin and North America. Despite the economic importance of the olive fly, the colonization route of this species is unclear. We used nuclear microsatellite markers and mitochondrial DNA to provide information about the population structure and invasion route of olive fly populations in Turkey, as representative of the Eastern Mediterranean region. Adult fly samples were collected from 38 sublocations covering all olive growing regions in Turkey. The simple sequence variability data revealed a significant genetic variability in olive fly populations and a certain degree of differentiation between Mediterranean and Aegean populations. Mediterranean populations harbor higher levels of microsatellite variation than Aegean populations, which points to the eastern part of the Mediterranean as the putative source of invasion. mtDNA results suggest olive flies from the western part of Turkey are closely related to Italo-Aegean flies of the Mediterranean basin and the olive fly populations have invaded the northern part of the Mediterranean basin through western Turkey. In addition, finding specific American haplotypes in high frequencies might indicate that Turkey is the possible source of American olive fly populations. In order to more precisely characterize the population structure and invasion routes of this organism, more DNA-based sequence analysis should be carried out worldwide.

## Introduction

The olive fly, *Bactrocera oleae* (Gmelin) (Diptera: Tephritidae), is a serious insect pest of olive crops worldwide. The distribution of this insect is limited to regions where cultivated and wild olive trees are grown. Although it is widespread throughout the Mediterranean basin and Middle East, the current distribution of the species includes Central and South Africa, California, and Central America [1], [2], [3]. The olive fly causes significant quantitative and qualitative loss in the production of table olives and oil. Its larvae are monophagous, feeding exclusively on wild and cultivated olive fruits. The adult female lays her eggs beneath the epidermis of ripening olive fruits; the hatched larvae feed inside the fruit by destroying the pulp, allowing entry of microbial infections. Feeding damage can cause premature fruit drop and reduces fruit quality for table olive and oil production. Damage has been estimated at 15% of total olive production, nearly USD 800 million/year [4]. Turkey, an important trading center for olive products for thousands of years, is the fifth leading olive producer in the world, with 8.2 × 10^5^ ha (data from FAO, 2010; http://faostat.fao.org).

A detailed understanding of the biology, genetic structure, and geographical variability of a given species is a prerequisite to designing effective quarantine, control, or eradication strategies [5]. Initial molecular studies of the olive fly were based on gel electrophoresis techniques and restricted to one or a few natural populations or their comparisons with laboratory colonies [6]–[12]. Recent advances in molecular technologies have provided new tools to monitor natural populations and their invasion pathways. Microsatellites, a nuclear co-dominant marker subject to Mendelian inheritance, display considerable polymorphisms due to variation in the number of repeat units, making them useful molecular markers for population studies [13]–[15]. Mitochondrial DNA (mtDNA) is an important molecular tool for reconstructing evolutionary events, such as identification of the region of origin of a species and the pathways of invasion and historical demography, and complements well with the information provided by microsatellite markers. The power of mtDNA analyses derives from its simple mode of inheritance (maternal and non-recombining), relatively high mutation rate, and the availability of comparative data with other species [16], [17]. The nuclear microsatellite markers of the olive fly have been developed [2], [18], [19] and the complete mtDNA sequence has been published [16]. Polymorphic microsatellite markers and mitochondrial DNA haplotypes have also been used to study genetic polymorphisms in other Tephritidae species such as *B. dorsalis* [17], [20], *B. tyroni* [21], [22], *B. cucurbitae* [23], [24], and *Ceratitis capitata* [25], [26], in order to understand evolutionary influences on invasive processes, and to identify routes of colonization.

Using microsatellite and mtDNA markers, genetic differentiation in different geographical populations and the invasive process of the olive fly were analyzed at the macrogeographical level. After examining the expansion and colonization history of the olive fly, 3 separate genetic groups, Pakistan, Africa, and the Mediterranean plus America, were revealed; Africa is suggested as the possible origin of this species [18], while the American samples seem to originate from the eastern Mediterranean [18], [27], [28]. Microsatellite markers revealed 3 subpopulations in the northern part of the Mediterranean basin: western (Iberian Peninsula), central (Greece-Italy), and eastern (Cyprus) [29], which was subsequently extended to Israel and California [27]. The results also indicated a westward expansion of the species associated with a gradual decrease in variability (expected heterozygosity, *H_e_*) from Cyprus to Portugal [29]. Westward expansion of the species, associated with a gradual decrease in variability, may have occurred concurrent with the introduction of the cultivated olive from its Levantine center of domestication to the Mediterranean basin [27], [29]. The invasion of olive flies across Europe is one hypothesis that has been proposed to explain the observed gradient of genetic variability across the Mediterranean area. An alternative interpretation, based upon another study by sequencing the whole mitochondrial genome of olive flies sampled from around the world [28] suggested an older origin, associated with the fragmentation of the wild olive host in different glacial refugia on this continent. The early co-history of the olive fly with its wild host was explained in detail in the same study. Besides these two main scenarios, the olive fly population of Tunisia was found genetically different from the populations of the northern shores of the Mediterranean basin [30]; therefore, expansion might occur from the southern to the northern coast of the Mediterranean basin.

The Eastern Mediterranean region is a putative invasion point for olive fly into the Mediterranean basin [29] and America [18], [27], [28]. Despite its importance for understanding the overall picture of genetic diversity and bio-invasion within this species, unfortunately, no detailed research has been carried out in this region. Genetic analysis of the olive fly bio-invasion process in this region can provide us a more complete understanding of the historical distribution, pattern of olive fly movements, and additional information for control of this important pest. The aims of this study were a) to investigate the population structure and genetic variability of different geographical populations of *B. oleae* in Turkey and b) to provide detailed information about the expansion and colonization history of the species. Field-collected populations of olive fly from 38 different sublocations, selected to be representative of the entire distribution area and covering the eastern to western parts of Turkey (from the far eastern point of Islahiye to the far western point of Gökçeada, covering a distance >1300 km) were analyzed for the 12 most polymorphic microsatellite loci known and their mtDNA haplotypes.

## Materials and Methods

### Ethics Statement

No specific permits were required for the described field studies for this wide spread agriculture pest. We confirm that the location is not privately owned or protected. The field studies did not involve endangered or protected species.

### Olive Fly Samples

**Figure 1:**
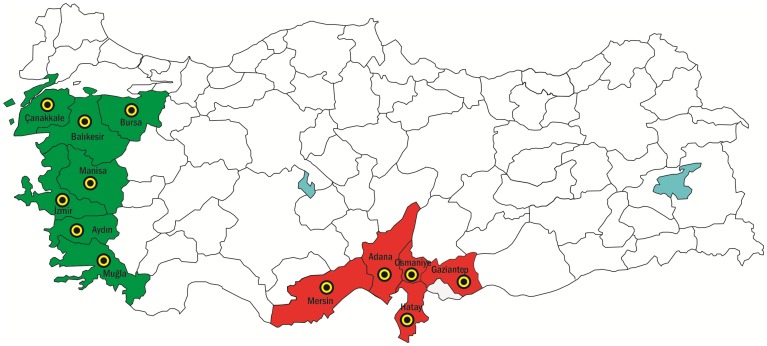
Distribution collection sites, green; indicates Aegean populations and red; indicates Mediterranean populations.

Olive fly samples were collected from egg-infested fruits in olive orchards during the harvest season in all major olive growing regions of Turkey in 2010. The sampled provinces included Çanakkale, Bursa, Balıkesir, Manisa İzmir, Aydın, and Muğla, in the Aegean region; Mersin, Adana, Osmaniye, Hatay, and Gaziantep in the Mediterranean region (Table 1 and Figure 1). From each province, 3 different sublocations (for Bursa and Çanakkale 4 different sublocations) were used as sampling sites (in total 38 different sublocations from 12 provinces). Different trees were sampled to limit sibling collections. Samples from each population were kept in separate cages to prevent mixing and incubated in the laboratory at 25°C until larvae emerged and developed to adulthood. Adult samples were frozen and stored at −80°C until use.

**Table 1:** *B. oleae* sampling locations.

Regions	Provinces	Sub-locations	Coordinates
**Aegean**	**Çanakkale**	Eceabat	40° 10.8′ N 26° 19.2′ E
		Geyikli	39° 48.0′ N 26° 10.8′ E
		Gökçeada	40° 12 0′N 25° 52.5′ E
		İntepe	40° 00.0′ N 26° 18.0′ E
	**Bursa**	Yalova	40° 39.0′ N 29° 16.2′ E
		Erdek	40° 25.2′ N 27° 46.8′ E
		Mudanya	40° 22.2′ N 28° 22.8′ E
		Gemlik	40° 25.8′ N 29° 09.0′ E
	**Balıkesir**	Küçükkuyu	39° 33.0′ N 26° 34.8′ E
		Zeytinli	39° 34.2′ N 26° 43.2′ E
		Edremit	39° 33.0′ N 26° 34.8′ E
	**Manisa**	Turgutlu	38° 30.0′ N 27° 42.0′ E
		Salihli	38° 28.2′ N 28° 09.0′ E
		Saruhanlı	38° 43.8′ N 27° 34.2′ E
	**İzmir**	Bornova	38° 27.0′ N 27° 13.2′ E
		Kemalpaşa	38° 25.2′ N 27° 25.2′ E
		Menemen	38° 36.0′ N 27° 03.0′ E
	**Aydın**	Çine	37° 37.2′ N 28° 03.0′ E
		Germencik	37° 52.2′ N 27° 34.8′ E
		İncirliova	37° 49.8′ N 27° 42.0′ E
	**Muğla**	Gökova	40° 46.2′ N 43° 37.8′ E
		Yerkesik	37° 07.8′ N 28° 16.2′ E
		Bayır	37° 19.8′ N 28° 06.0′ E
**Mediterranean**	**Mersin**	Silifke	39° 34.2′ N 26° 43.2′ E
		Tarsus	36° 55.8′ N 34° 55.8′ E
		Mezitli	36° 49.2′ N 34° 46.2′ E
	**Adana**	Kozan	37° 27.0′ N 35° 48.0′ E
		Kürkçüler	37° 16.2′ N 35° 37.8′ E
		Karaisalı	37° 13.8′ N 35° 03.0′ E
	**Osmaniye**	Cevdetiye	37° 07.2′ N 36° 22.2′ E
		Kadirli	37° 22.2′ N 36° 04.2′ E
		Toprakkale	37° 04.2′ N 36° 07.8′ E
	**Hatay**	Samandağ	36° 04.8′ N 35° 58.8′ E
		Altınözü	36° 06.0′ N 36° 13.8′ E
		Antakya	36° 13.2′ N 39° 09.0′ E
	**Gaziantep**	Nurdağı	37° 10.1′ N 36° 44.2′ E
		Zincirli	37° 07.2′ N 36° 39.0′ E
		Islahiye	36° 13.2′ N 39° 09.0′ E

### Amplification of Microsatellite Loci

The 12 most polymorphic microsatellites primers (listed in Table 2) were tested and chosen from the 20 previously characterized microsatellite primers for this organism [2], [18], [29]. Primers were labeled by using 3 different fluorescent dyes, HEX, 6-FAM, and NED. From each sublocation, 10 adult individuals (total 380) were used for microsatellite analysis. Total DNA was extracted by the Lifton method [31]. Amplification of microsatellite loci was performed as described [18], [29]. After PCR, 1 µL of each reaction was visualized on 2% agarose gel and the products were analyzed on an ABI PRISM 3100 Genetic Analyzer (Applied Biosystems). Electropherograms were manually checked with the Applied Biosystems Peak Scanner program (http://www.appliedbiosystems.com) and recorded.

**Table 2:** Microsatellite loci.

Locus	Motif	Primer sequences (5^′^–3^′^)	Allele Size	Reference
Bo-D37	(CA)_7_CG(CA)_3_	**F**:ATAGGCATTGGCAGCGAAG**R**:CACAGTGGGCCGAAATCAC	172–182	[2]
Bo-D42	(CA)_10_GA(CA)_2_	**F**: CAGAGCATCTCGCTTTGG **R**: TCAACAATCCCAGCAAAATC	136–172	[29]
Bo-D51	(GT)_12_	**F**: TGGAATGCGCTATTTTGTTG **R**: ACTCGTATATACGTACATGG	140–170	[29]
Bo-D52	(GA)_14_	**F**: CGACTTGAAGGACAATTGG **R**: GGCGTGAGTAGTTTCTATAAGC	111–130	[2]
Bomic15	(AC)_8_	**F**: CAGCCAACCAGTCAACC **R**: GTTTGGCTGAAATGGCAGTCC	118–142	[18]
Bo-D49	(GT)_13_	**F**: TCGCCTCTTACCTCACAACC **R**: ACCATCCTTAGTCAGCACAGTC	157–185	[29]
Bo-D54	(GT)_17_	**F**: CTGACTTCTTGCTTTACACG **R**: CAGCTTATCTGCTTTAAGTGC	123–163	[2]
Bo-D48	(CA)_13_	**F**: GCCATGAATGCAGACCAC **R**: CCTATTCAAATGCACGCAAAAC	153–165	[29]
Boms59	TGTA(TG)_10_	**F**:AGCGCTTACATAAATATAGCTAC**R**: TCCCCGTAAAGCCATAAAGTC	158–174	[29]
Boms61	A_11_CA_11_CATCACA_4_GA_2_GA_8_	**F**: ACTGAAATGCAGCTTATTGGC **R**: ATGAAGCGACTGGCACGAG	175–187	[29]
Bo-D53	(GT)_10_	**F**: TGAAGGTGATGAATGAAAGC **R**: GGAATGACTGTGAGCAAGC	143–163	[29]
Boms31	(GT)4GC(GT)6GC(GT)2	**F**: TGCTTGAGTTGCTCGTTGG **R**: GCCGCATGACATAAAGAATCG	144–170	[29]

### Amplification of Mitochondrial Haplotypes

From each sublocation, 7 individual flies (total 266) were used for sequencing the most polymorphic 574 bp of the first subunit of the mitochondrial NADH dehydrogenase (ND1) gene. The experiment was performed as described [18] with modifications. In order to amplify the corresponding region, a new primer pair (Bo3EDF: 5′-AGTCAATGAGCTTGAACAAGCATGTG-3′ and Bo4EDR: 5′-AGGTATTCCTCAACCTTTTTGTGAC-3′) was used. These primers were designed using the published mitochondrial genome of *B. oleae,* GenBank accession no. AY210703. After PCR, the products were visualized and isolated from 1.2% agarose gel by Qiagen QIAquick PCR purification kit according to the manufacturer’s instructions and sequenced directly by using BoND1F (5′-TTTAGTTGCTTGGTTGTGTATTCC3′; obtained from [18]) and Bo4EDR primers. Sequencing was performed on an Applied Biosystems A3100 automated DNA sequencer. Electropherograms were checked manually.

### Data Analysis

#### Microsatellite data analysis

Genetic polymorphism within populations was determined as the mean number of alleles per locus (*n_a_*), effective number of alleles (*n_e_*), and observed (*H_o_*) and expected heterozygosity (*H_e_*) using POPGENE version 1.31 [32]. The same program was used to calculate the genetic distance values according to Nei [33] and agreement of genotypic frequencies with Hardy-Weinberg equilibrium (HWE) in populations with chi-square (χ^2^) and likelihood ratio G^2^; corrections were performed by applying Bonferroni correction for multiple comparisons [34]. Statistical comparisons were performed using PAST (version 2.07) [35]. Linkage disequilibrium, significance of association between genotypes at all pairs of loci, was tested with POPGENE and GENETIX software [36]. The phylogenetic relationships between populations based on genetic distances were depicted by a neighbor-joining tree, constructed using POPULATIONS version 1.2.32 [37] with bootstrap value = 100. The Analysis of Molecular Variance (AMOVA) test was performed for 2 different population groupings (east-west) in ARLEQUIN v. 3.5 [38]. Genetic variation was partitioned into 3 levels; among populations, among populations within individuals, and within individuals. Relationships between genetic and geographic distances among populations, isolation by distance, were assessed by the Mantels test [39]. Geographic distances between localities were estimated using the web site www.googleearth.com. STRUCTURE software 2.1 [40], [41] was used to investigate the number of possible genetic clusters (or distinct groups) of *B. oleae* in Turkey. This program assumes a model in which there are K populations, each of which is characterized by a set of allele frequencies at each locus. To determine the most likely number of clusters (K) in our population samples, we used different values of K ranging from 1 to 12. The degrees of genetic differentiation among populations were analyzed as pairwise *F_ST_* values using FSTAT software 2.9.3 [42]. This program was also used to calculate allelic richness (AR). Gene flow (*N_e_*m, the number of effective migrants per generation) between geographic regions was calculated from *F_ST_* (averaged over the 12 loci) according to the formula: *N_e_*m = [(1/F*_ST_*)−1)]/4 [43]. Finally, BOTTLENECK software [44] was used to infer demographic expansion/contraction in each population.

#### mtDNA data analysis

DNA sequences were edited and verified as follows. First, primer sequences were removed from the raw files. The 574-bp portion of ND1 mtDNA sequences from 266 *B. oleae* samples were aligned with and without the previously published mitochondrial ND1 gene sequences (GenBank accession numbers AY998304 to AY998325 [18] and GU108459 to GU108478 [28]) using CLUSTALW [45]. Descriptive statistics (number of variable sites, number of haplotypes, haplotype diversity, average number of nucleotide differences between haplotypes) were calculated in Dnasp (ver. 5.0) [46]. Median-joining networks of haplotypes, including previously identified haplotypes, were constructed using NETWORK (ver. 4.6) [47], [48].

## Results

To unravel the variability and colonization process of *B. oleae* in Turkey, genetic polymorphism of natural olive fly populations was studied by using nuclear DNA (microsatellite) and mitochondrial markers.

### Microsatellite Variability

The analysis of 12 microsatellite loci in 380 flies, captured from 38 sublocations in 12 provinces, showed high levels of variability (Table S1). The number of alleles per locus varied from 5 (Boms61) to 23 (Bo-D54), with an overall mean of 13.92±4.94. The mean number of effective alleles detected per locus was 4.48±1.14, while the highest number was observed at Bomic15 with 7.63; the lowest value was at Bo-D37 with 3.34. Polymorphism was also determined with regard to the level of heterozygosity. Observed (*H_o_*) and expected (*H_e_*) heterozygosity values were 0.78±0.08 and 0.77±0.05, respectively, when averaged over loci. *H_o_* values ranged from 0.69 (Boms61 and Bo-D53) to 0.91 (Bomic15); *H_e_* values ranged from 0.70 (Bo-D37) to 0.87 (Bomic15). Geographical distribution of microsatellite alleles by population for each locus was also determined. The number of detected alleles varied from 4 to 16 in all populations. The highest number of private alleles (up to 5) was present at Bo-D51 locus and no private allele was detected at Bo-D48 and Boms61 loci.

After sequential Bonferroni correction [34], all sampled populations were confirmed to be in HWE at all loci according to χ^2^ and G^2^ criteria (at P<0.05). The average *F_ST_* over all loci and the number of effective migrants per generation, *N_e_*m, were 0.03 and 8.36 respectively. No linkage disequilibrium was detected between genotypes at all loci; all were considered independent.

Table 3 shows the overall level of variability relative to the 12 loci in the 12 analyzed olive fly populations. The mean number of alleles (*n*_a_) ranged from 7.58 (Adana) to 9.50 (Mersin) and allelic richness (AR) from 7.05 (Bursa) to 8.88 (Mersin). The amount of genetic variation seemed to be homogeneously distributed among different populations, considering mean heterozygosity estimates of *H*_o_ and *H*_e_. The observed and expected heterozygosity values varied from 0.75 (Mugla) to 0.82 (Aydın) and 0.74 (Bursa, Manisa, and Adana) to 0.78 (Mersin, Osmaniye, and Gaziantep). However, mean number of effective alleles (*n_e_*), number of private alleles (*n_p_*), and frequency of private alleles (A*_p_*) were found to be significantly different between two regions (P<0.05, Mann-Whitney U test). The results were: *n_e_* = 4.01, *n_p_* = 1, A*_p_* = 0.01 in Aegean populations and *n_e_* = 4.36, *n_p_* = 3.4, A*_p_* = 0.03 in the Mediterranean populations. In total, 24 new private alleles were determined in low frequencies (encountered only once or twice), 17 of them in the Mediterranean region. Although the number of private alleles is dependent on sample size [49], [50], the Bursa, Çanakkale, and Manisa populations had no private alleles. The Mersin population presented the highest level of genetic diversity including mean and effective number of alleles, allelic richness, private alleles with high frequency (≥0.04), and expected heterozygosity among studied populations. In comparison with the other populations, lower variability values were observed in the Bursa population.

**Table 3:** Genetic variability in field-collected samples of *B. oleae* from different geographical regions of Turkey.

Regions	Location	N	*n_a_*	AR	*n_e_*	*n_p_*	A*_p_*	*H_o_*	*H_e_*
**Aegean**	Çanakkale	40	8.00	7.14	3.98	0	0	0.76	0.75
	Bursa	40	8.00	7.05	3.91	0	0	0.76	0.74
	Balıkesir	30	8.00	7.58	3.99	3	0.03	0.78	0.75
	Manisa	30	8.08	7.64	3.92	0	0	0.78	0.74
	İzmir	30	8.25	7.81	3.91	1	0.01	0.78	0.75
	Aydın	30	8.25	7.86	4.25	1	0.01	0.82	0.77
	Muğla	30	7.75	7.50	4.13	2	0.02	0.75	0.76
	**Mean**		**8.05**	**7.51**	**4.01**	**1**	**0.01**	**0.78**	**0.75**
**Mediterranean**	Mersin	30	9.50	8.88	4.71	5	0.04	0.79	0.78
	Adana	30	7.58	7.20	3.93	3	0.03	0.77	0.74
	Osmaniye	30	9.00	8.44	4.48	3	0.03	0.77	0.78
	Hatay	30	8.00	7.52	4.22	3	0.03	0.77	0.76
	Gaziantep	30	9.00	8.42	4.49	3	0.02	0.80	0.78
	**Mean**		**8.61**	**8.09**	**4.36**	**3.4**	**0.03**	**0.78**	**0.77**
	**Test of Significance**		ns	ns	*	*	*	ns	ns

N, number of flies analyzed; *n* ***_a_***_,_ mean number of alleles; AR: allelic richness; *n_e_*, mean number of effective alleles; *n_p_*, number of private alleles; A***_p_***, frequency of private alleles; *H* ***_o_***, mean observed heterozygosity; *H_e_*, mean expected heterozygosity; ns, not significant; *, significant at P<0.05.

Genetic relationships among populations were quantified by pairwise *F*_ST_ (Table 4). The *F*_ST_ values ranged from −0.00197 (between Muğla-Çanakkale) to 0.05146 (between Aydın-Adana). Analysis of significance of pairwise *F*_ST_ values among all samples indicated the possibility of grouping in 2 subpopulations. Mediterranean populations were generally significantly different from, although at different probability levels, populations of the Aegean region. However, the genetic differentiation within regions was low. This lack of differentiation can be explained by smooth topography, continuous plant cultivation (including plant exchanges), and extensive olive trades among provinces in these regions. Considering the genetic variability among Mediterranean and Aegean populations, the data were analyzed by separating the populations into 2 main geographical groups and the mean pairwise *F*_ST_ value was 0.01379 (*P*<0.001). The distance between these 2 geographical regions is greater than 700 km. The level of gene flow, considering this distance, for olive fly populations in Turkey can be considered an important factor influencing the shape of the genetic structure of this organism.

**Table 4:** The range of *F*_ST_ values between olive fly populations and the significance of population differentiation estimated by *F*_ST_ values *P<0.05; **P<0.001; ***P<0.0001.

Regions	Population	Çanakkale	Bursa	Balıkesir	Manisa	İzmir	Aydın	Muğla	Mersin	Adana	Osmaniye	Hatay	Gaziantep
**Aegean**	**Çanakkale**	–											
	**Bursa**	0.00345	–										
	**Balıkesir**	0.00442	0.01827***	–									
	**Manisa**	0.00162	0.0102	0.01388	–								
	**İzmir**	0.00972*	0.02423**	0.01293*	0.00353	–							
	**Aydın**	0.0086**	0.01947***	0.00819	0.00947	0.00509	–						
	**Muğla**	−0.00197	0.00121	0.00222	−0.0039	0.00441	0.00286	–					
**Mediterranean**	**Mersin**	0.00324*	0.01004*	0.0273***	0.01444*	0.02049**	0.03082***	0.00277	–				
	**Adana**	0.0233***	0.03339***	0.05037***	0.03281***	0.04616***	0.05146***	0.02965***	0.01244	–			
	**Osmaniye**	0.01357*	0.0242**	0.033***	0.01697	0.02075*	0.02151**	0.00792	0.00519	0.01362	–		
	**Hatay**	0.01024*	0.0084	0.03237***	0.01796*	0.02742**	0.02914***	0.00694*	0.00396	0.01742***	0.00448	–	
	**Gaziantep**	0.0059	0.01647***	0.02358***	0.01226	0.01789**	0.01635**	0.00041	0.00863	0.01126	0.00763	0.00798	–

**Figure 2:**
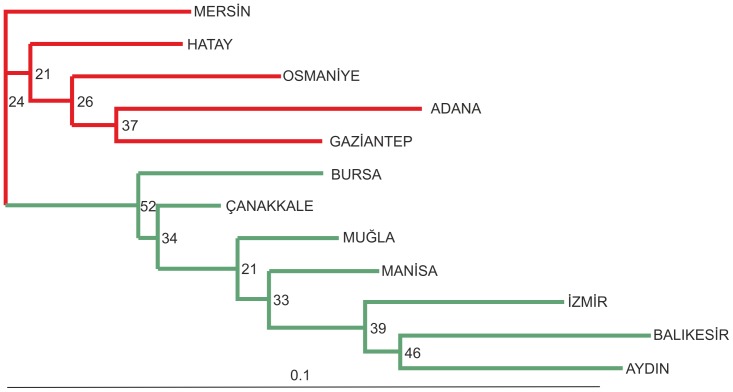
Unrooted neighbor-joining tree of 12 *B. oleae* populations using 12 polymorphic microsatellite loci. Numbers at each node indicate bootstrap values. Branches are color coded: red for Mediterranean populations, green for Aegean populations.

The unrooted NJ tree of 12 populations based on genetic distances presented in Figure 2. Most of the branches had low bootstrap values. The observed tree is not inconsistent with, but nor does it conclusively demonstrate, the Aegean and Mediterranean populations forming two separate lineages.

To further analyze the structure of olive fly populations in Turkey, Factorial Correspondence Analysis was performed. This analysis also showed the same topology as the unrooted NJ tree with 2 main clusters: Mediterranean and Aegean (Figure S1).

To analyze the isolation by distance among populations, the Mantel test was performed using microsatellite markers (Figure S2). Results indicated the presence of a correlation between genetic and geographic distances (Spearman Rank correlation coefficient r = 0.4738; Mantel P<0.002).

Tests of homogeneity among the populations were performed using AMOVA. For this purpose, the population groups were chosen according to the 2 major geographical areas clustered in the population tree: the Aegean and Mediterranean regions of Turkey. The results are summarized in Table 5. AMOVA confirmed a regional structure among groups (*P*<0.0019). The main contribution to genetic variance was due to variation within individuals while little genetic variation was attributable to the variation among populations/within groups.

**Table 5:** Analysis of molecular variance (AMOVA).

Structure	Source of Variation	%Total variance	Fixation indices
Two major regions	Among groups	1.24	*F_CT_* = 0.01238
	Among populations/within groups	0.91	*F_SC_* = 0.00924
	Within individuals	97.85	*F_ST = _*0.01379

The genetic structure of the populations was also analyzed based on microsatellites using STRUCTURE software (Figure S3). A low level of differentiation is depicted by this analysis. Although the number of clusters (K) varied from K = 1 to 12, no significant genetic differentiation was observed. We also tested the hypothesis of a recent bottleneck based on the TPM. The bottleneck test, with a mode shift in allele frequency classes, attributed an L-shaped distribution to all populations, consistent with normal frequency class distribution ranges (P>0.05).

### mtDNA Analysis

To explore the invasion history of this species from the eastern Mediterranean to Europe, a portion of the mitochondrial ND1 gene was used to investigate genetic diversity, haplotype phylogeny, and demographic history of 12 olive fly populations from all olive cultivating areas in Turkey. Forty-four haplotypes, variant sequence forms, were observed and given haplotype designations in 266 individuals. Thirty-five of these haplotypes were unique to Turkey and 9 of them were shared with previously identified haplotypes [18], [28]. The availability of such unique haplotype variants provided us a powerful tool for resolving questions relating to various aspects of the invasion process of olive fly populations in our region. Newly determined sequences were deposited in GenBank under accession numbers JX271833-JX271867 (available after Jan 01, 2013).

The haplotypes identified in our study were genetically similar with 1 to 8 substitutions between pairs (no deletions or insertions were detected). Basic descriptive indices of genetic diversity for each population are presented in Table 6. Haplotype diversity (h) ranged from 0.49524 (Hatay) to 0.93651 (Bursa) with the average overall value of 0.812±0.019, similar to the h value (0.79±0.04) [18] in the Mediterranean basin. Sequence divergence (π) among the haplotypes changed from 0.0009 (Hatay) to 0.0048 (Bursa) with the overall mean of 0.003±0.0001. Even though different numbers of flies were sampled from the Bursa and Çanakkale populations, the number of haplotypes from each location was 3 (Hatay) to 15 (Bursa). The mean number of haplotypes (Hp), h and π values for each region, were Hp = 9.57, h = 0.854, and π = 0.0038 in Aegean populations; Hp = 6.6, h = 0.6504, and π = 0.002 in Mediterranean populations. Almost all populations showed high levels of genetic variability, except for the Hatay population. In 574 bp, there were 38 polymorphic sites (6.62% of total length), 17 of these sites were singletons, the mutation being present in a single haplotype sequence, and 21 of them were parsimony informative.

**Table 6:** Haplotype diversity in olive fly populations.

Regions	Population	N	Hp	h	π
**Aegean**	Çanakkale	28	9	0.8148	0.0036
	Bursa	28	15	0.9365	0.0048
	Balıkesir	21	8	0.8476	0.0036
	Manisa	21	8	0.8238	0.0039
	İzmir	21	10	0.9	0.0037
	Aydın	21	11	0.8904	0.0042
	Muğla	21	6	0.7619	0.0030
	**Mean**		**9.57**	**0.854**	**0.0038**
**Mediterranean**	Mersin	21	9	0.6809	0.0025
	Adana	21	8	0.7238	0.0023
	Osmaniye	21	7	0.7428	0.0025
	Hatay	21	3	0.4952	0.0009
	Gaziantep	21	6	0.6095	0.002
	**Mean**		**6.6**	**0.6504**	**0.002**

N: number of flies analyzed, Hp: number of haplotypes, h: haplotype diversity, π: nucleotide diversity.

The list of identical haplotypes from this study and previous studies are presented in Table S2. Although fewer flies were used (45 flies), 11 different haplotypes from 8 locations were identified in the Mediterranean basin (from Israel-Haifa to Portugal-Paradale) [18]. Our results showed that the western region of Turkey and the European part of the Mediterranean basin are most closely related, sharing 6 out of 10 haplotypes. The number of these common haplotypes was 1/4 from Portugal, 2/3 from France, 3/4 from Italy, and all haplotypes from Greece, Israel, and previous reports from Turkey. In America, 5 different haplotypes were identified [18] and 4 of them, except for haplotype L (found in Ensenada-Mexico and Burguret Forest-Kenya), were observed in our study. Two of these haplotypes (haplotypes N and O) were specific to the American continent and we observed both haplotypes with high frequencies with no regional specification (Table S2 and S3). Ten haplotypes were reported from Pakistan and Africa, the source of Mediterranean populations [18]. We found just one African haplotype reported previously [18] in Turkey, haplotype A (Table S2). In another study, 4 previously known haplotypes, H1, H2, H4, and H17 were identified by using 11 samples from the Mediterranean basin [28]. Three of them, H1, H2, and H4, were observed in our study; however, we did not observe haplotype H17 (found in Paradale-Portugal and Sig City-Algeria) (Table S3).

The mitochondrial haplotypes were differentially distributed among the Turkish olive fly populations. Table S3 lists the haplotypes, frequencies, and distributions among the populations of *B. oleae*. Four haplotypes, H1, H2, H4, and H8, were found at higher frequencies and were the most common, widely distributed variants, comprising 75.1% of all 266 sequences in Turkey. Haplotypes H1 and H2 were shared by all studied populations and they seem to be fixed in Turkey. Haplotype H1, comprising 37.7% of all 266 sequences, was a common (dominant) haplotype in our region. Sixty percent of the eastern Mediterranean and 40% of the Aegean regions are grouped in H1. The second-most common haplotypes H2 and H8 were found at a frequency of 13.1%. In haplotype H2, 52% of Mediterranean and 48% of Aegean samples were grouped together. However, samples from the Aegean region had a higher frequency (with 83%) of haplotype H8. H4, which is the fourth most common haplotype with a frequency of 11.2%, contained only samples from western Turkey.

A rough association was observed between geographical source of individuals and genetic groups of haplotypes. Ten and twenty-seven haplotypes were specific to the Mediterranean and Aegean regions, respectively. However, each of the remaining 7 haplotypes was observed in more than one region. Haplotype 23 was found on the small island Gokceada, a sublocation of Çanakkale, and seems to be unique to this island.

**Figure 3:**
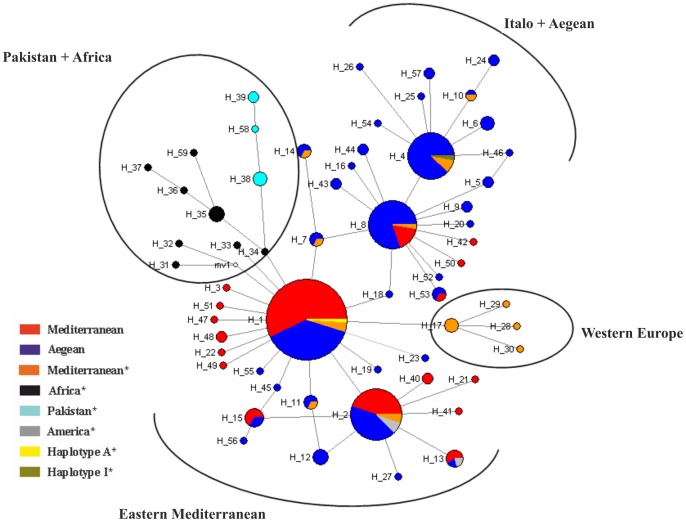
Mitochondrial haplotype network. Haplotype numbers and their distributions by region are presented in Table S2 and S3. The areas of the circles are proportional to the number of samples sharing each haplotype. Empty circles represent haplotypes not observed in the sample. Haplotypes are colored by region. *Data obtained from [18] and [28].

The relationships between mitochondrial haplotypes identified in our dataset, together with previously published worldwide data (GenBank accession numbers AY998304-AY998325 and GU108459-GU108478), were defined by a haplotype network (Figure 3). The haplotype-based network analyses allowed us to better understand the important aspects of genetic structure and phylogenetic relationships of olive fly populations in Turkey and worldwide. The network revealed the existence of 3 separate groups in the Mediterranean basin: eastern Mediterranean-America, Italo-Aegean (including western Turkey), and western Europe (or the Iberian clade). Consistent with previous studies [18], [28], Pakistan and African haplotypes are well structured and differentiated from the Mediterranean haplotypes, showing the strong phylogeographic structure of the populations. Although one of the haplotypes (H7) from Paradale-Portugal was found in 2 different regions of Turkey (Table S3), haplotypes from Portugal, Italy, and Algeria (haplotypes 17, 28, 29, and 30) formed the western European cluster. Four main haplotypes of Turkey (H1, H2, H4, and H8) and previously identified haplotypes were positioned in the network and the remaining haplotypes were found generally at lower frequencies and connecting to these haplotypes through few mutations. The sequence of the most common haplotype, H1, shared 100% identity with the previously identified haplotypeA [18]. In his study, this haplotype was found with the overall frequency of 0.30%; in our study, the frequency of this haplotype was slightly higher (37.7%). Combining our results with his study, H1 seems to be a common haplotype for the olive fly in the eastern Mediterranean and America. Later, another identical haplotype of A was found in a sample from Morocco [28]. The second most common haplotype, H2, has the same sequence as haplotypeN, which was only identified in Mexico, the USA [18], and USA -haplotypeOroville- [28]. HaplotypeO (in H13) has only been reported in the USA [18] and is connected to H2 by one mutation. This haplotype also seems to be specific to the American continent. Another common haplotype, H8, is connected to H1 by two mutations and is predominant in western samples; only 17% of this haplotype was formed by Mediterranean sequences. H4 contains only samples from western Turkey and has the same sequence as previously identified haplotypes from Bari and Vaggia (Italy) [28]; haplotype I is the most common and widely distributed allele in Europe [18]. HaplotypeJ (in H10) was found in Italy [18] and was connected to H4 by one mutation. This haplotype and other haplotypes (except for H8) diverge by one or two mutational steps and were all derived from western Turkey; it seems that H4 is specific to this and the Italo-Aegean region.

## Discussion

In this study, we used both mitochondrial and nuclear DNA markers to unravel the patterns of genetic differentiation and the potential invasive route of *B. oleae* from the eastern Mediterranean to Europe. Turkey is an important part of the eastern Mediterranean region, but previously relatively little was known mainly because of the limited number of analyzed specimens [18], [29].

### Population Structure of B. oleae in Turkey

The simple sequence variability data reveal two main findings: (i) a level of genetic variability is present in the olive fly populations in Turkey; (ii) a certain degree of differentiation between Mediterranean and Aegean populations might indicate the possible expansion of this fly from east to west.

According to some authors [51], [52], a correlation is expected between the level of genetic variability and the degree of environmental diversity of a species. The potential for ecological heterogeneity to increase genetic diversity, and perhaps divergence, has been suggested [53], [54]. In our study, *B. oleae*, a strictly monophagous species, met such expectations over a wide area. We observed a high level of genetic variability among the olive fly populations by using 12 polymorphic microsatellite markers and samples collected from different regions of Turkey. This result is consistent with the literature; previous population studies [18], [27], [29], [30] of this organism reported a high degree of genetic variability on a regional geographic scale, which seems to be characteristic of this species. One of the main causes of this high genetic variation is the length of time that has elapsed since the species became established in the eastern Mediterranean. Domestication of wild olive trees took place in the Near East around the 4th millennium BC [28], [55], [56], and archeological ruins indicate that the olive was processed in Anatolia for the first time around 3^th^–2^nd^ millennium BC [57]. However, it is possible that most of the evolutionary history of the species has in fact taken place on wild olives before the domestication of cultivated olives [58]. The oldest olive seed was found in Anatolia (specifically in Tarsus-Mersin region) and dated from the Neolithic age, 8000 to 5000 BC [59]. The elevated effective size of the populations is another factor contributing to genetic variability, since olive groves cover wide expanses in our region and olive fly populations are expected to remain large over time.

Cluster analysis points to the existence of two subpopulations in Turkey: Mediterranean and Aegean. The results of the NJ tree, although with low bootstrap values (see Figure 2) and PCA (Figure S1), support the existence of these major subpopulation groups. The Mantel test performed with microsatellite markers revealed a positive correlation between geographic distances and genetic distances among populations, indicating isolation by distance.

Three main hypotheses have been proposed to describe the colonization and expansion history of olive fly populations towards the European parts of the Mediterranean basin [27]–[30] but the underlying population dispersion processes remain partially unclear. Our SSR data, coupled with the presence of a high number of low-frequency alleles, seem to suggest the westward expansion of this fly from the eastern Mediterranean to the northern Mediterranean basin [27], [29]. The Mediterranean populations are the most polymorphic with higher genetic variability values and a greater number of private alleles. Briefly, as stated before [27], [29], the east-to-west colonization of the olive fly is accompanied by a gradient loss of polymorphism and linked to the westward expansion of olive cultivation. Furthermore, according to archeological data, olives were transported from Anatolia to the Greek islands and Greece [60].

The observed high level of gene flow (*N*em = 8.36) between populations does not seem to be enough to homogenize the olive fly populations in Turkey. SSR markers revealed genetic variability and differentiation among olive fly populations in Turkey. There are 3 possible reasons for this differentiation. First, continuous host resources, the absence of natural barriers to gene flow, and appropriate climatic conditions are favorable to the olive fly at the Syrian border of Turkey. This may form a natural route for population dispersal from the Middle East to the Mediterranean region of Turkey, i.e. from south to north or vice versa. Different authors have reported the high dispersal capacity for geographical expansion of the olive fly [3], [61], [62]. Second, limited human-mediated effects (such as new olive plantations, transportation, and trade) and less continuous distribution of olives from east to west might be another factor. Third, local variation in selection intensity (for agricultural purposes) might be strong enough to maintain variation between regions. The western regions of Turkey have large areas of olive cultivation and intense selection pressures have being applied to these populations through long-standing eradication programs.

In our study, the Mersin population showed the highest genetic variability in Turkey. There are 2 possible reasons for this high variability; First, Mersin is one of the first regions where olive cultivation was systematically initiated in Anatolia between 2000–1200 BC [57], [60]. The second reason for this variability might be that the region is the closest point between Turkey and Cyprus, among the first Mediterranean islands where olive trees were systematically cultivated. An intensive olive trade has been carried out with this island and other Mediterranean countries such as Egypt and the Greek islands since historical times via a seaport in this city [60].

### Haplotype Analysis and the Population Structure of B. oleae in Turkey

Our mtDNA results provide valuable information for understanding olive fly invasion from the eastern Mediterranean to Europe. We have 3 main conclusions; (i) olive flies from western Turkey are most closely related to Italo-Aegean flies of the Mediterranean basin; (ii) olive fly populations invaded the northern Mediterranean basin through western Turkey; (iii) and Turkey is the possible source of American olive fly populations.

Unlike the results obtained from microsatellites, mitochondrial data indicates a higher level of mean genetic diversity (number of haplotypes, haplotype diversity, and nucleotide diversity) in the Aegean than in the Mediterranean region (Table 6). One reason for this difference is a single haplotype, H1, predominates in southeastern Turkey. This discrepancy may also be the result of different evolutionary patterns of both markers; mtDNA is maternally transmitted in animals, it evolves quite slowly in comparison to microsatellites, and it is more prone to genetic drift [63].

Population structure of the Mediterranean basin has been studied previously by using mtDNA sequences [18], but failed to identify population-level genetic differentiation. A clear phylogenetic separation between eastern and central/western Mediterranean populations was reported after sequencing the whole mitochondrial genome in a limited number of flies [28]. Our network analysis, similar to the findings of a recent study [64], indicates 3 main groups in the Mediterranean basin; eastern Mediterranean-America, Italo-Aegean-western Turkey, and western Europe (or Iberian clade). Although no clear split has been observed between the eastern Mediterranean-America and Italo-Aegean populations in our study, Turkey seems to contain both subpopulations (Figure 3). Six European-specific haplotypes (H4, H7, H8, H10, H11 and H14 in Table S2 and S3) out of 10 previously identified haplotypes [18] from Greece to France are found specifically in western Turkey, although at different frequencies (H8 is also found in the Mediterranean region). These common haplotypes are distributed to different torsos in the Network (Figure 3). It seems that the border of the Italo-Aegean population extends from the western coast of Turkey to France, from which 2 haplotypes (out of 3) were identified in Turkey. In addition, it was mentioned by [60] that Phokaians transferred cultivated Anatolian olive varieties from western Turkey to Marseille (France) in 600 BC. These kinds of olive transportations during history may explain the observation of common haplotypes between central Europe and western Turkey. However, more detailed sequence analysis should be performed on samples from central Europe to clarify this issue.

The Western Europe group in the network contains H17, H28, H29, and H30 haplotypes (Figure 3) mainly identified in western Europe and northwestern Africa (Table S3). Arabian olive varieties were introduced to the Iberian Peninsula after the 8^th^ century AC [29]. This might explain the separation of the Western Europe from the Anatolian groups. H4, a specific haplotype for the Aegean region, contains the common European haplotype I (Table S2) and is connected to European-specific haplotype J, which is one mutational step away (Figure 3). H8 predominates in western samples and seems to be a transient torso between H1 and H4.

H1, the only shared haplotype from Africa, is the most common and widespread variant especially in southeastern populations of Turkey. H2, being a specific haplotype for American samples, is found at very high frequency in all studied regions. Another specific American haplotype is H13, which is differentiated from H2 by one mutational step and is observed in 2 different regions of Turkey. Previous studies [18], [27] supported the Middle Eastern origin of American populations. Determination of the wide distribution and high frequencies of these specific haplotypes in our study indicates the possible origin of Turkey for the American populations. However, it should be kept in mind, limited number of sequences was available from this continent.

Based on Network analysis and the distribution of haplotypes, an east (Mediterranean) to west (Aegean) invasion route is inferred, as suggested by our SSR data. The fact that the Aegean region shares many haplotypes (6/10) with eastern and central Europe indicates the species may have invaded Europe through western Turkey, i.e. olive fly expansion occurred via the northern part of the Mediterranean basin. This observed westward expansion of the species supports a previously expressed hypothesis [29].

It is always possible that additional haplotypes might exist in natural olive fly populations in the Eastern Mediterranean-America and Italo-Aegean regions; however, these would be limited to the tips or fringes of the network, considering the large number of flies and wide collection area used in this study. Direct analysis of DNA sequence-based haplotypes from other parts of genomes might help us to understand the movement of this pest from the Middle East to Europe.

The olive fruit fly is the most important pest of wild and cultivated olives. Laboratory and field observations have shown that female *B. oleae* exhibited strong ovipositional preferences for certain varieties of cultivated olives [65], [66], [67], [68]. The resulting larvae performed better in preferred olive varieties than in lesser or non-preferred varieties. These varietal differences in larval performance in olive have implications for the success of an invasion and range expansion process, which effects population growth and dynamics of the organism during its adaptation to a new environment. Once introduced into an area the fly’s establishment time could be effected by the olive variety present [68]. More information about cultivar susceptibility to olive fruit fly will also help us to clarify olive fly distribution and colonization process in future.

## Supporting Information

Figure S1 **The result of Factorial Correspondence Analysis.**
(TIF)

Figure S2 **Geographic distance plotted against genetic distance (as FST/(1−FST )) calculated between samples of flies based on Mantel’s test.** b) Distances (km) between locations.
(TIF)

Figure S3 **Results of Structure analyses from K = 2 to K = 12.**
(TIF)

Table S1 **Microsatellite variability in Turkey.** N: number of flies used; *n_a_*: number of actual alleles; *n_e_*: number of effective alleles; *H_o_*: observed heterozygosity; *H_e_*: expected heterozygosity.
(DOC)

Table S2 **List of identical haplotypes from previous studies.**
(DOC)

Table S3 **Distribution and frequency of different mitochondrial haplotypes in populations of** ***B. oleae*** **in Turkey.** The identical haplotypes were given in Table S2. The haplotype locations are given in parentheses. *data obtained from [18], ** from [28]. Mer: Mersin; Ada: Adana; Osm: Osmaniye; Hat: Hatay; Gantp: Gaziantep; Man: Manisa; İzm: İzmir; Muğ: Muğla; Ayd: Aydın; Çnkl: Çanakkale; Besr: Balıkesir; Bur: Bursa.
(DOC)
