# Supplementary figures and images for: The Genetic Polymorphisms and Colonization Process of Olive Fly Populations in Turkey
Source: PLoS One. 2013 Feb 14;8(2):e56067. doi: 10.1371/journal.pone.0056067 (PMC3573072; doi:10.1371/journal.pone.0056067)

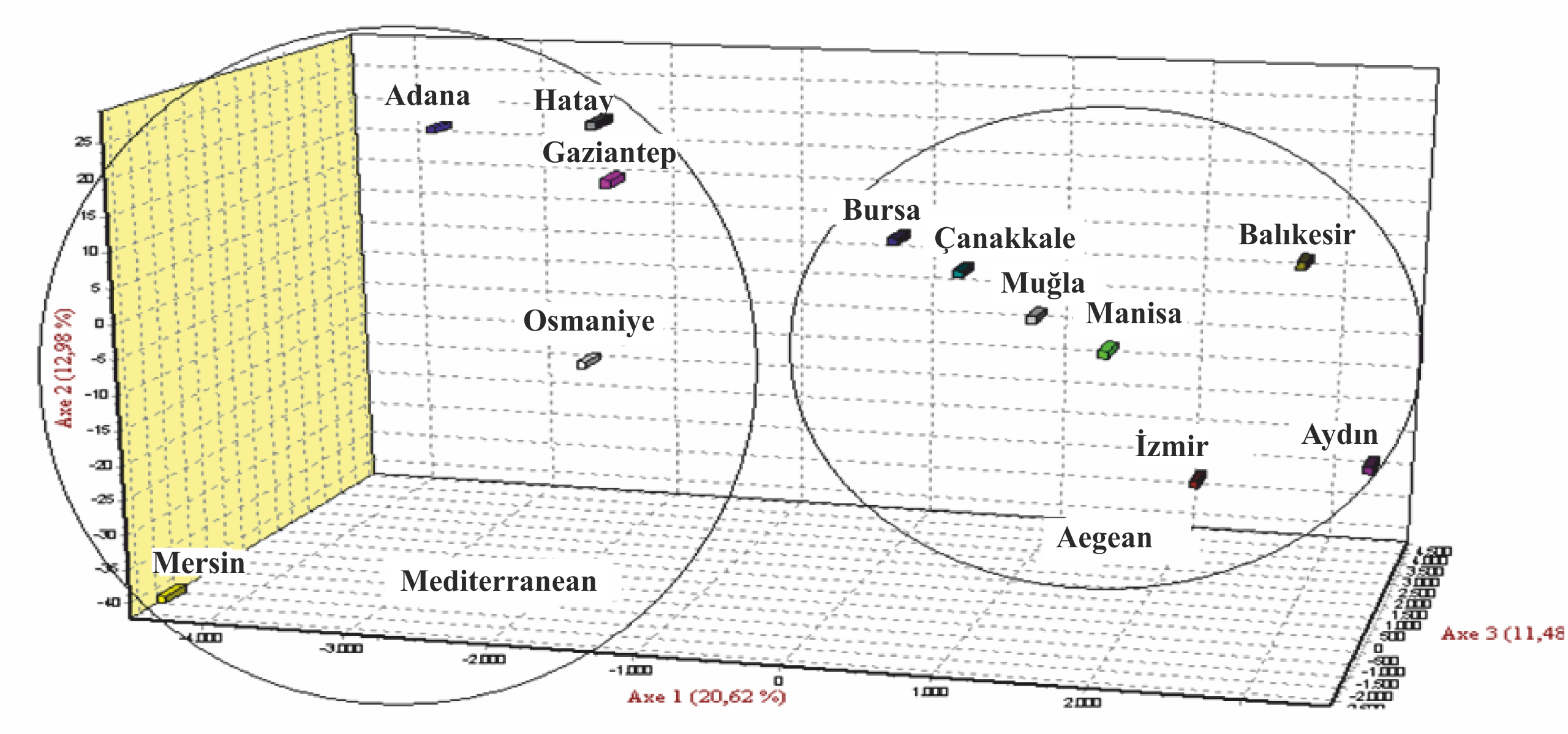

Supplement: Figure S1 — The result of Factorial Correspondence Analysis. (TIF) [file pone.0056067.s001.tif]

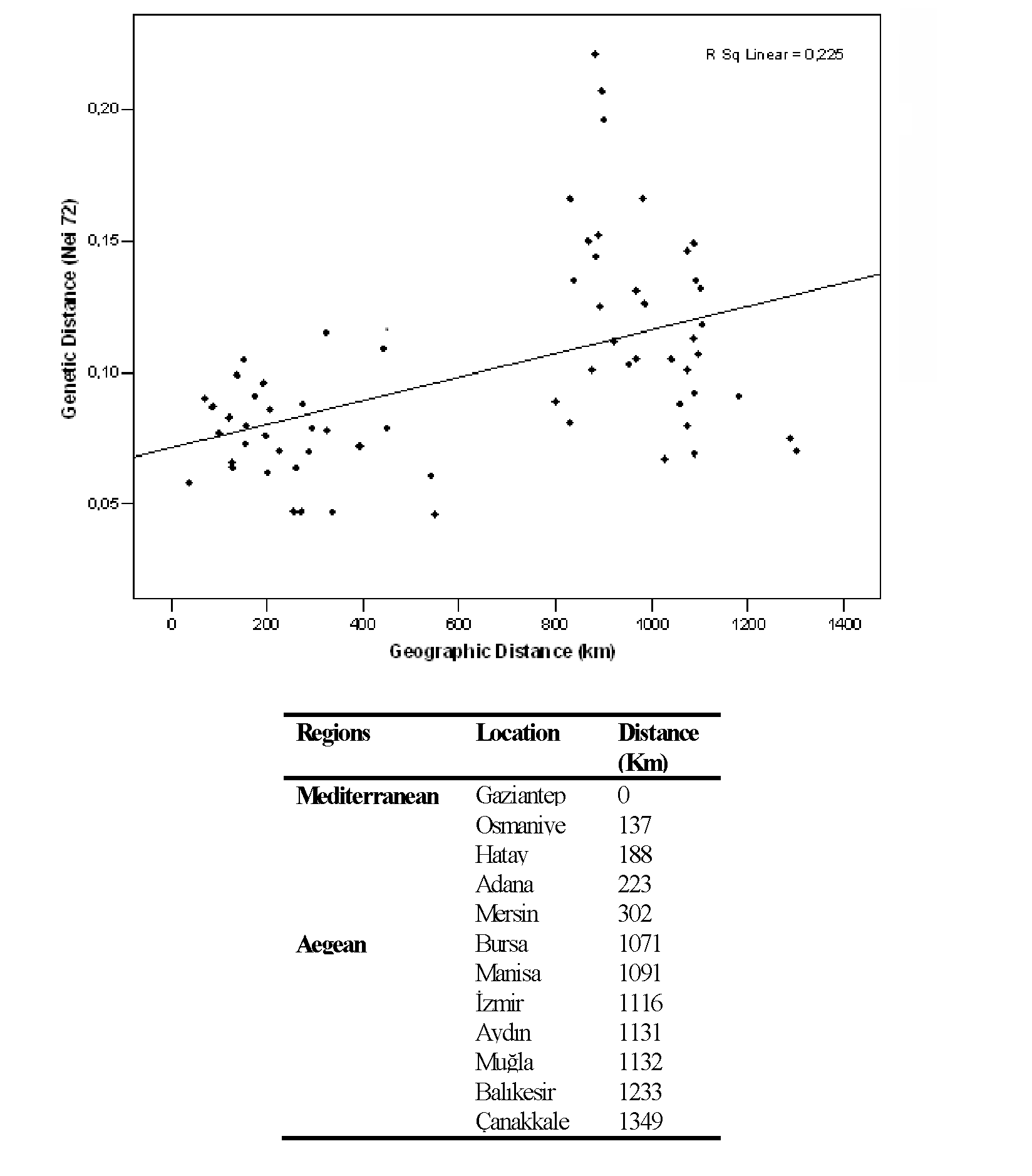

Supplement: Figure S2 — Geographic distance plotted against genetic distance (as FST/(1−FST )) calculated between samples of flies based on Mantel’s test. b) Distances (km) between locations. (TIF) [file pone.0056067.s002.tif]

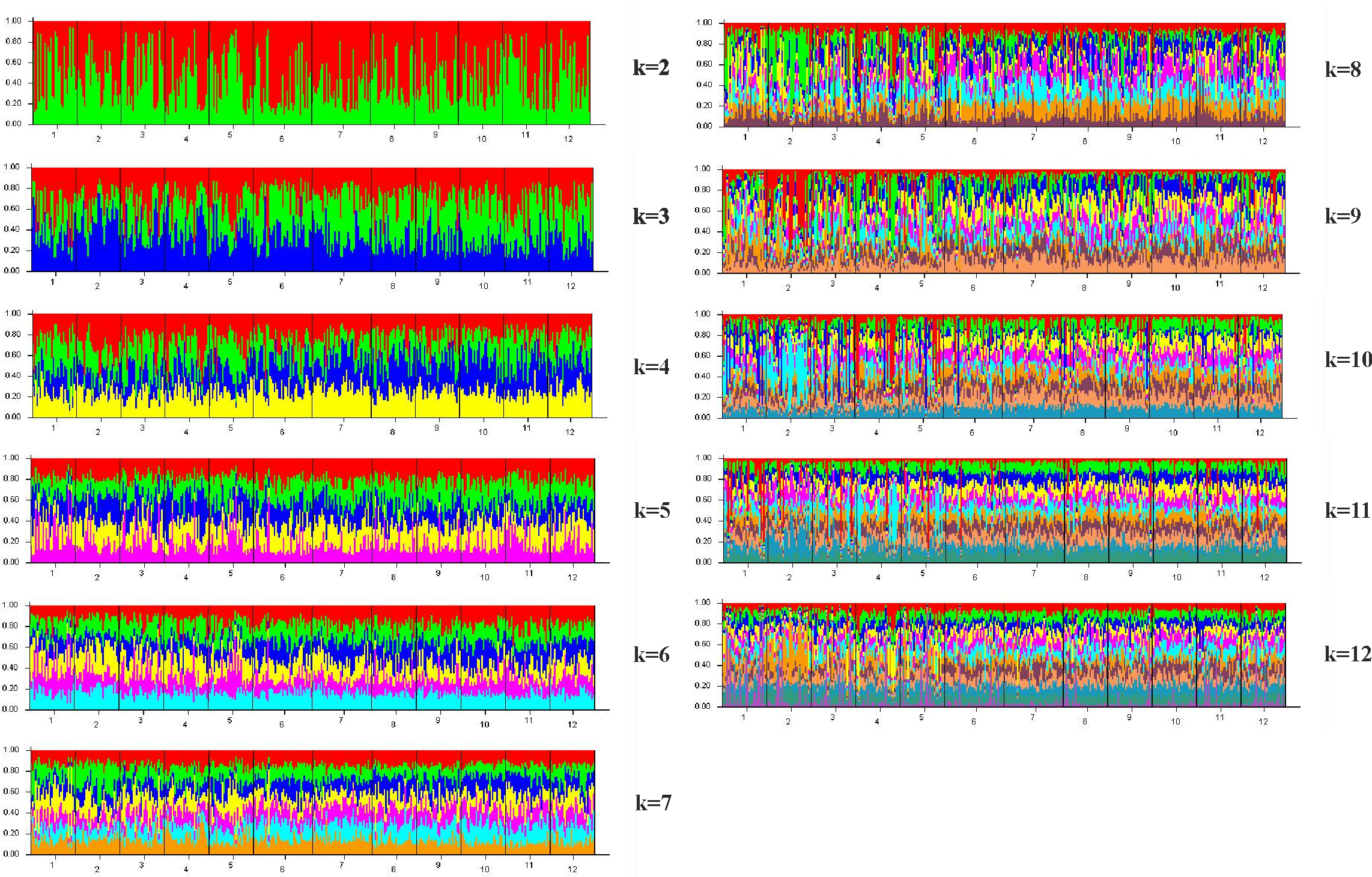

Supplement: Figure S3 — Results of Structure analyses from K = 2 to K = 12. (TIF) [file pone.0056067.s003.tif]
