# Supplementary material for: The Genetic Polymorphisms and Colonization Process of Olive Fly Populations in Turkey
Source: PLoS One. 2013 Feb 14;8(2):e56067. doi: 10.1371/journal.pone.0056067 (PMC3573072; doi:10.1371/journal.pone.0056067)
Supplement: Table S1 — Microsatellite variability in Turkey. N: number of flies used; na: number of actual alleles; ne: number of effective alleles; Ho: observed heterozygosity; He: expected heterozygosity. (DOC) [file pone.0056067.s004.doc]

**Table S1.** Microsatellite variability in Turkey. N: number of flies used; *n_a_*: number of actual alleles; *n_e_*: number of effective alleles; *H_o_*: observed heterozygosity; *H_e_*: expected heterozygosity

|  | **Locus** | **N** | ***n_a_*** | ***n_e_*** | ***H_o_*** | ***H_e_*** |
| --- | --- | --- | --- | --- | --- | --- |
|  | Bo-D52 | 374 | 15.00 | 5.13 | 0.82 | 0.81 |
|  | Bo-D37 | 374 | 11.00 | 3.34 | 0.74 | 0.70 |
|  | Bo-D42 | 374 | 17.00 | 4.42 | 0.77 | 0.77 |
|  | Bo-D51 | 373 | 17.00 | 4.16 | 0.70 | 0.76 |
|  | Bomic15 | 363 | 18.00 | 7.63 | 0.91 | 0.87 |
|  | Bo-D49 | 363 | 18.00 | 5.12 | 0.90 | 0.81 |
|  | Bo-D54 | 363 | 23.00 | 3.82 | 0.72 | 0.74 |
|  | Bo-D48 | 360 | 11.00 | 4.36 | 0.79 | 0.77 |
|  | Boms59 | 373 | 11.00 | 3.89 | 0.81 | 0.74 |
|  | Boms61 | 373 | 5.00 | 3.97 | 0.69 | 0.75 |
|  | Bo-D53 | 369 | 9.00 | 3.48 | 0.69 | 0.71 |
|  | Boms31 | 372 | 12.00 | 4.50 | 0.78 | 0.78 |
| **Mean** |  | 369 | **13.92** | **4.48** | **0.78** | **0.77** |
| **SD** |  |  | 4.94 | 1.14 | 0.08 | 0.05 |
