# Supplementary material for: The Genetic Polymorphisms and Colonization Process of Olive Fly Populations in Turkey
Source: PLoS One. 2013 Feb 14;8(2):e56067. doi: 10.1371/journal.pone.0056067 (PMC3573072; doi:10.1371/journal.pone.0056067)
Supplement: Table S2 — List of identical haplotypes from previous studies. (DOC) [file pone.0056067.s005.doc]

**Table S2.** List of identical haplotypes from previous studies

| **Haplotype Name** | **Identified Location** | **GeneBank Accession Number** | **Source** | **Identical haplotypes in our study** |
| --- | --- | --- | --- | --- |
| Haplotype A | Kenya-Burguret Forest | AY998304 | [18] | H1 |
|  | California-Santa Barbara |  |  |  |
|  | İsrael-Haifa |  |  |  |
|  | Mexico-Ensenada |  |  |  |
|  | Turkey-Osmaniye |  |  |  |
| Haplotype-Morroco | Morroco- Marrakesh | GU108474 | [28] |  |
| Haplotype-Catania | Italy-Catania | GU108471 | [28] |  |
| Haplotype-Haifa2 | Israel-Haifa | GU108472 | [28] |  |
| Haplotype-Osmaniye1 | Turkey-Osmaniye | GU108465 | [28] |  |
| Haplotype N | Mexico-Ensenada | AY998317 | [18] | H2 |
|  | California-Santa Barbara |  |  |  |
| Haplotype-Oroville | California- Oroville | GU108475 | [28] |  |
| Haplotype-Osmaniye2 | Turkey-Osmaniye | GU108461 | [28] |  |
| Haplotype-Haifa1 | İsrael-Haifa | GU108460 | [28] |  |
| Haplotype-SantaBarbara | California- SantaBarbara | GU108479 | [28] |  |
| Haplotype I | France-Les Matelles | AY998312 | [18] | H4 |
|  | Italy- Montecucco |  |  |  |
|  | Italy- Portoferraio |  |  |  |
|  | Italy- Catania |  |  |  |
|  | Greece- Athens |  |  |  |
|  | Mexico-Ensenada |  |  |  |
| Haplotype-Bari1 | Italy-Bari | GU108459 | [28] |  |
| Haplotype-Bari2 | Italy-Bari | GU108460 | [28] |  |
| Haplotype-Vaggia | Italy-Vaggia | GU108464 | [28] |  |
| Haplotype F | Portugal-Paradela | AY998309 | [18] | H7 |
|  | Italy-Portoferraio |  |  |  |
| Haplotype H | France-Les Matelles | AY998311 | [18] | H8 |
|  | İsrael-Haifa |  |  |  |
| Haplotype J | Italy-Montecucco | AY998313 | [18] | H10 |
|  | Italy-Catania |  |  |  |
| Haplotype K | Greece- Athens | AY998314 | [18] | H11 |
| Haplotype O | California-Santa Barbara | AY998318 | [18] | H13 |
| Haplotype G | Greece-Athens | AY998310 | [18] | H14 |
