# Supplementary material for: The Genetic Polymorphisms and Colonization Process of Olive Fly Populations in Turkey
Source: PLoS One. 2013 Feb 14;8(2):e56067. doi: 10.1371/journal.pone.0056067 (PMC3573072; doi:10.1371/journal.pone.0056067)
Supplement: Table S3 — Distribution and frequency of different mitochondrial haplotypes in populations of B. oleae in Turkey. The identical haplotypes were given in Table S2. The haplotype locations are given in parentheses. *data obtained from [18], ** from [28]. Mer: Mersin; Ada: Adana; Osm: Osmaniye; Hat: Hatay; Gantp: Gaziantep; Man: Manisa; İzm: İzmir; Muğ: Muğla; Ayd: Aydın; Çnkl: Çanakkale; Besr: Balıkesir; Bur: Bursa. (DOC) [file pone.0056067.s006.doc]

**Table S3.** Distribution and frequency of different mitochondrial haplotypes in populations of *B. oleae* in Turkey. The identical haplotypes were given in Table S2. The haplotype locations are given in parentheses. *data obtained from [18], ** from [28]. Mer: Mersin; Ada: Adana; Osm: Osmaniye; Hat: Hatay; Gantp: Gaziantep; Man: Manisa; İzm: İzmir; Muğ: Muğla; Ayd: Aydın; Çnkl: Çanakkale; Besr: Balıkesir; Bur: Bursa

| **Aegean** | | | | | | | | **Mediterranean** | | | | |
| --- | --- | --- | --- | --- | --- | --- | --- | --- | --- | --- | --- | --- |
| **Haplotype/ Location** | **Çnkl** | **Bur** | **Besr** | **Man** | **İzm** | **Ayd** | **Muğ** | **Mer** | **Ada** | **Osm** | **Hat** | **Gantp** |
| H1 | 8 | 5 | 4 | 7 | 5 | 2 | 9 | 12 | 11 | 10 | 14 | 13 |
| H2 | 1 | 3 | 3 | 4 | 3 | 2 | 1 | 2 | 3 | 4 | 6 | 3 |
| H3 |  |  |  |  |  |  |  |  |  |  | 1 |  |
| H4 | 7 | 3 | 7 | 5 | 1 | 4 | 3 |  |  |  |  |  |
| H5 | 1 |  | 1 |  |  |  |  |  |  |  |  |  |
| H6 | 1 |  | 1 | 1 |  |  |  |  |  |  |  |  |
| H7 | 1 |  |  |  | 1 |  |  |  |  |  |  |  |
| H8 | 7 | 4 | 2 | 1 | 4 | 6 | 5 | 1 | 2 | 3 |  |  |
| H9 |  |  | 2 |  |  |  |  |  |  |  |  |  |
| H10 |  |  |  |  |  | 1 |  |  |  |  |  |  |
| H11 |  | 1 |  |  |  | 1 |  |  |  |  |  |  |
| H12 |  | 2 |  | 1 |  | 1 |  |  |  |  |  |  |
| H13 |  |  |  |  |  | 1 |  | 1 | 1 | 1 |  |  |
| H14 |  |  |  |  | 1 | 1 |  |  |  |  |  |  |
| H15 |  | 1 |  |  |  | 1 |  | 1 | 1 | 1 |  | 1 |
| H16 |  |  |  |  |  | 1 |  |  |  |  |  |  |
| H17 HaplotypeB* (Paradale-Portugal, Les Matteles-France) and HaplotypeAlg** (Sig city-Algeria) |  |  |  |  |  |  |  |  |  |  |  |  |
| H18 |  | 1 |  |  |  |  |  |  |  |  |  |  |
| H19 |  | 1 |  |  |  |  |  |  |  |  |  |  |
| H20 |  | 1 |  |  |  |  |  |  |  |  |  |  |
| H21 |  | 1 |  |  |  |  |  |  |  | 1 |  |  |
| H22 |  |  |  |  |  |  |  |  |  | 1 |  |  |
| H23 | 1 |  |  |  |  |  |  |  |  |  |  |  |
| H24 | 1 | 1 |  |  |  |  |  |  |  |  |  |  |
| H25 | 1 | 1 |  |  |  |  |  |  |  |  |  |  |
| H26 |  | 1 |  |  |  |  |  |  |  |  |  |  |
| H27 | 1 |  |  |  |  |  |  |  |  |  |  |  |
| H28 HaplotypeC*  (Paradale-Portugal) |  |  |  |  |  |  |  |  |  |  |  |  |
| H29 HaplotypeD*  (Paradale-Portugal) |  |  |  |  |  |  |  |  |  |  |  |  |
| H30 HaplotypeE* (Catania-Italy) |  |  |  |  |  |  |  |  |  |  |  |  |
| H31 HaplotypeL* (Burguret fst-Kenya, Ensenada-Mexico) |  |  |  |  |  |  |  |  |  |  |  |  |
| H32 HaplotypeM*  (Burguret fst-Kenya) |  |  |  |  |  |  |  |  |  |  |  |  |
| H33 HaplotypeP*  (Burguret fst-Kenya) |  |  |  |  |  |  |  |  |  |  |  |  |
| H34 HaplotypeQ*  (Burguret fst-Kenya) |  |  |  |  |  |  |  |  |  |  |  |  |
| H35 HaplotypeR* (Burguret fst-Kenya, Paarl Mountain-S.Africa) |  |  |  |  |  |  |  |  |  |  |  |  |
| H36 HaplotypeS*  (Paarl Mountain-S.Africa) |  |  |  |  |  |  |  |  |  |  |  |  |
| H37 HaplotypeT*  (Paarl Mountain-S.Africa) |  |  |  |  |  |  |  |  |  |  |  |  |
| H38 HaplotypeU* (Cherat-Pakistan, Malakand-Pakistan) |  |  |  |  |  |  |  |  |  |  |  |  |
| H39 HaplotypeV* (Cherat-Pakistan, Malakand-Pakistan) |  |  |  |  |  |  |  |  |  |  |  |  |
| H40 |  |  |  |  |  |  |  |  |  |  |  | 2 |
| H41 |  |  |  |  |  |  |  |  |  |  |  | 1 |
| H42 |  |  |  |  |  |  |  |  |  |  |  | 1 |
| H43 |  |  |  |  | 2 |  |  |  |  |  |  |  |
| H44 |  |  |  |  | 2 |  |  |  |  |  |  |  |
| H45 |  |  |  |  | 1 |  |  |  |  |  |  |  |
| H46 |  |  |  |  | 1 |  |  |  |  |  |  |  |
| H47 |  |  |  |  |  |  |  |  | 1 |  |  |  |
| H48 |  |  |  |  |  |  |  | 1 | 1 |  |  |  |
| H49 |  |  |  |  |  |  |  |  | 1 |  |  |  |
| H50 |  |  |  |  |  |  |  | 1 |  |  |  |  |
| H51 |  |  |  |  |  |  |  | 1 |  |  |  |  |
| H52 |  | 1 |  |  |  |  |  |  |  |  |  |  |
| H53 |  |  |  |  |  |  | 2 | 1 |  |  |  |  |
| H54 |  |  |  |  |  |  | 1 |  |  |  |  |  |
| H55 |  |  |  | 1 |  |  |  |  |  |  |  |  |
| H56 |  |  |  | 1 |  |  |  |  |  |  |  |  |
| H57 |  | 2 |  |  |  |  |  |  |  |  |  |  |
| H58 Haplotype Mlknd**-(Cherat-Pakistan, Malakand-Pakistan) |  |  |  |  |  |  |  |  |  |  |  |  |
| H59 HaplotypePaarl Mnt**( Paarl Mountain-S.Africa) |  |  |  |  |  |  |  |  |  |  |  |  |
